# Study protocol of the X:IT II - a school-based smoking preventive intervention

**DOI:** 10.1186/s12889-019-6805-2

**Published:** 2019-05-02

**Authors:** Lotus Sofie Bast, Pernille Due, Stine Glenstrup Lauemøller, Niels Them Kjær, Tenna Christiansen, Anette Andersen

**Affiliations:** 1 National Institute of Public Health, University of Southern Denmark, Studiestreade 6, 1455 Copenhagen, Denmark; 2 Danish Cancer Society, Strandboulevarden 49, 2100 Copenhagen, Denmark

**Keywords:** Adolescents, Smoking prevention, Tobacco prevention, School intervention, Differential effect, Socioeconomic groups

## Abstract

**Background:**

The X:IT intervention, conducted in 2010 to 2013, showed overall smoking preventive effect. However, parts of the intervention appeared less appealing to children from families with lower socioeconomic backgrounds. Therefore, the intervention components were modified and an evaluation of the amended intervention X:IT II is needed to show the effect of this revised intervention and whether children from different social backgrounds benefits equally from the current intervention.

**Methods:**

Main intervention components are smoke free schools, a curricular component, and parental involvement (smoke free agreements and talks about tobacco). Components have been revised from the first version; 1) previously, schools should be smoke free on the school ground and were encouraged to hide smoking so that it wasn’t visible to pupils from the school ground. Now they are encouraged to tighten the rules so that no pupils or teachers smoke during the school day, no matter where they are; 2) the specifically developed educational material (Up in Smoke) has been revised so that all materials are online and all texts has a ARI; 3) the parental involvement is now targeted multiple groups of parents, e.g. parents that are smokers, and parents of children that smoke. Language used is simpler and the website for parents presents very specific examples.

X:IT is implemented in 46 Danish public schools from fall 2017 until summer 2020. Data is collected through electronic questionnaires to students and coordinators four times (fall 2017, spring/summer 2018, 2019 and 2020). Further, qualitative interviews and observations are conducted.

**Discussion:**

Prevalence of smoking among Danish adolescents is high compared to other Nordic countries and there is social inequality in smoking, leaving individuals from the lowest social backgrounds at higher risk. Although there has been an overall decline in smoking among Danish adolescents over the last decades, a recent levelling of this development indicates an urgent need for smoking prevention in Denmark. The X:IT intervention has the potential to prevent uptake of smoking among adolescents. However, there is a particular need for evaluating the effectiveness of the revised X:IT intervention, X:IT II, with focus on the effect across socioeconomic groups of adolescents.

**Trial registration:**

Current Controlled Trials ISRCTN31292019, date of registration 24/10/2017. Retrospectively registered.

## Background

In Denmark, as well as in most Westernized countries, smoking is more prevalent among adolescents from lower socioeconomic positions (SEP) [1]. While 8 % of 15-year old Danish adolescents from higher SEP smoke (daily or less), up to 24% of adolescents whose parents receive welfare benefits are smokers [2]. Furthermore, adolescents from low SEP seem less likely to quit smoking in adulthood [3]. Public health interventions to reduce risk behaviours (e.g. smoking) should aim at being equally effective across socioeconomic groups or at being more effective among individuals from low SEP [4].

Interventions addressing health and health behaviours in childhood or adolescence have the potential to prevent or decrease socioeconomic inequalities in health behaviours later in life [5]. On the other hand, interventions themselves may generate or increase socioeconomic inequalities in health behaviours, e.g. in smoking [6]. This is problematic to the extent that interventions may be less effective or have counter-effects in the most at risk groups, often low SEP. Socially differential effects of interventions may arise at a number of points in the implementation process of an intervention, including intervention efficacy, service provision or access to materials and tools, uptake, and compliance [7]. Most intervention studies on smoking have not addressed these aspects and therefore, the mechanisms behind any inequality or lack of inequality of the effect of interventions are unknown. It has been argued that interventions targeting individual aspects related to behavioral change are more likely to widen social inequalities compared to interventions targeting the environment, because the interventions targeting the individual directly require more effort and resources from each individual [8].

Schools are regarded suitable settings for prevention activities for children and adolescents, as the school setting offers the possibility to reach almost all within the relevant age group and across different socioeconomic positions [9, 10]. In the past decades, a large number of school-based smoking prevention programs have been implemented internationally, but evaluations have shown contrasting results regarding their effectiveness [11]. Generally, strategies using comprehensive components, including environmental strategies, have been found more effective than exclusively information based interventions, which have shown limited or no effect [12, 13]. The history of school based smoking prevention in Denmark includes participation in the international intervention study “The European Smoking Prevention Framework Approach” (ESFA). Unfortunately, there was no effect of ESFA in Denmark [14]. Two other school-based interventions in Denmark, “Smoke-free classes” and “Tackling” also reported no effect [15, 16].

In 2010, the Danish Cancer Society launched the X:IT intervention. The effect of the intervention was tested in a randomized controlled trial, testing the implementation in 53 Danish schools compared to 41 control schools from 2010 to 2013 [17]. X:IT turned out to reduce smoking uptake with up to 25% among participants after the first year of study [18]. However, the qualitative process evaluation showed that some of the intervention components were less appealing to children from lower socioeconomic positions [19], indicating that X:IT in the first version might have limited appeal to the group of adolescents from lower SEP. Therefore, the intervention components have been modified and an evaluation of this revised intervention X:IT II is needed to show whether pupils from different social backgrounds benefits equally from the intervention. This paper presents the modified X:IT II intervention and its intervention components as well as the design of the effect and process evaluation of X:IT II.

### The X:IT trial

The first X:IT study was a large-scale cluster randomized controlled trial conducted in 94 schools testing the effectiveness of the X:IT intervention among 13- to 15-year-olds [17].

The intervention was developed and implemented by the Danish Cancer Society. The Centre for Intervention Research was responsible for thorough scientific evaluation of implementation and effect of the intervention [18–21]. The X:IT intervention was developed using the Intervention Mapping Protocol as a systematic planning tool [22] and the Theory of Triadic Influences as theoretical framework [23].

The X:IT intervention targeted all students attending grade 7 to 9 (13- to 15-year-olds) in the intervention schools and X:IT included three main intervention components:

- smoke free school grounds
- parental involvement comprising two dimensions – (a) smoke free contract between the student and an adult person, preferably a parent, (b) smoke free dialogues between student and parents/adults
- a smoke free curriculum based on self-efficacy training and appraisal of outcome expectancies

The intervention was implemented from autumn 2010 until spring 2013 [17]. The effect evaluation showed the intervention to be effective in achieving its pre-specified aim of reducing smoking by 25% after the first year of intervention; the odds ratio for smoking among students attending an intervention school compared to control schools was 0.61 (95% CI: 0.45–0.82) [18]. Unfortunately, we saw a decrease in implementation over time; hence we were unable to show any effect after the second year [24]. Thorough examinations of the implementation process showed the importance of the degree of implementation for the intervention effects. Compared to students who were not exposed to the intervention, these analyses showed a stepwise increase in the effect of the intervention from OR 0.65 (CI: 0.43–0.97) for uptake of smoking for students exposed to medium implementation to OR 0.44 (CI: 0.32–0.68) for students exposed to full implementation of the intervention after the first year. Low implementation of the intervention did not significantly influence the smoking uptake among adolescents [20]. Further, subgroup analyses showed the importance of sufficient implementation of each of the three main intervention components [25].

As part of a thorough process evaluation of the X:IT intervention, a qualitative study among parents and children from low socioeconomic backgrounds participating in the X:IT intervention was conducted by The Danish Cancer Society [19]. The evaluation pointed at some important flaws in the applicability of the X:IT intervention for this group of adolescents and parents. The evaluation suggested that parents from low social class perceived the language used in the parental component as too academic. Parents who smoked wished for practical guides showing how to back up their children to stay smoke free [19]. Also, the educational material used in the X:IT intervention was criticized for using language which was challenging for many children from low social class background, as they are facing a risk of reading or literacy difficulties. Hence, there was a need to improve the X:IT components and test the reach and effect of a revised intervention among adolescents and parents from low social background before recommending national scale-up of the intervention.

## Methods and design

### Setting

Denmark has 98 municipalities with an average 55,000 inhabitants. There is approximately one school per 5000 inhabitants in a municipality. The Danish public school consists of grade 0 (preschool class) and grade 1–9. All 10 years are mandatory education. Danish children start school the year they turn 6. Children who start together in the same class at grade 0 will often belong to the same class/group of children through all ten years of schooling. In Denmark, children are not postponed or moved forward educationally due to academic skills or achievements, as is the case in many other countries. There is a limit of 28 children per class. Schools with grade 7–9 students usually have 2–4 parallel tracks. There is no grouping by ability in the Danish schools i.e. all children have joint lecturing. Among Danish children 85% attend public schools. The schools’ catchment area almost always comprises a wide distribution of socioeconomic background circumstances of students.

Parents have an important position at Danish schools. All schools are headed by a School Board, which is the highest deciding body at the school. The School Board has 5–7 parent representatives, two pupil members, and two staff members. The principal does not have a voting position, but is secretariat for the Board. All parents may be elected members of the School Board.

### The evaluation of X:IT II

The X:IT II intervention consists of the same three main intervention components, however in a revised form as described in the following.

#### Intervention components

##### Smoke free school grounds (smoke free school time)

Compared to most countries in the Western world, Denmark has had a very lenient smoking policy. Cigarette prices are relatively low and the first law restricting smoking in public places was adopted in 2007. In August 2012 smoking was fully banned for students, employees and visitors at Danish school grounds. This law was passed during the period of the first evaluation of X:IT. Therefore, the smoke free school ground component was revised accordingly. In the X:IT II intervention, the rules for smoke free schools are even firmer than the national law; here schools are encouraged to secure that there is no smoking anywhere at the school ground, or outside the school ground, during school hours. In Denmark, this is known as ´Smoke free school time´. The smoke free school time applies for students as well as teachers, other employees and visitors. Previous studies showed that students exposed to teacher smoking during the school day were more susceptible to smoke themselves [26–28].

##### Parental involvement

Two other Nordic studies have successfully used smoke free contracts between pupils and parents [29, 30]. In the Swedish study, the smoke free contract seemed to reduce the smoking prevalence by almost 50% among 14- to 15-year-olds [30]. Signing a smoke free contract is a manifestation of an active choice of non-smoking. When signing the contract, the pupil promises to stay smoke free for the following year. One of the parents, or another significant adult, co-signs the contract. The adult hereby commit to support the adolescent’s choice of staying smoke free and also to have a smoke free dialogue with their child. Having a smoke free dialogue involves that the adults clearly distance themselves from adolescent smoking, and asks about the child’s thoughts and experiences with tobacco.

In X:IT II, the smoke free contract and the smoke free dialogue have been revised. The material addressing parents, i.e. the homepage, leaflets, and the smoke-free contract, have been revised in order to tailor the language and information specifically for parents from lower SEP. The new material is now targeting various groups of parents: smoking parents, non-smoking parents, and parents with smoking children. The material has been revised to make the communication more unequivocal, relevant, and easier to read. For example, the material previously called ‘Smoke free dialogue’ is now called ‘Chat on tobacco’. The homepage (http://www.snakomtobak.dk/, In English: Chat on Tobacco) has become interactive and visually improved by use of videos, quizzes, dilemma games, and pictures. Further, the smoke free contract is renamed to “smoke free agreement” in order to make it more informal. As in the first version of X:IT, in X:IT II students with a signed smoke free agreement are able to win a prize [17].

The teachers present the X:IT II intervention to the parents at parent meetings at the beginning of each school year.

##### Smoke free curriculum

Generally, there is no strong evidence for the effectiveness of school-based programs that provide information-giving curricula only. However, information-giving programs seem to be effective if included in multi-model programs [13]. Programs based on social influence approaches which include: correcting adolescents’ perceptive overestimation of smoking prevalence; recognizing high-risk situations; increasing awareness of media, peer and family influences; teaching and practicing refusal skills; and making public commitments not to smoke, have been shown to be effective [13, 31].

The educational material ‘Gå op i Røg’ (Up in Smoke) is based on the knowledge above. The program has been developed to target students at grade 7 to 9 (age 13–15 years), and includes eight lessons a year for three years. It can be used in several different subjects (cross-curricular). All texts have been made available online and the automated readability index (ARI) is lowered. The general readability has been improved, i.e. a glossary appears when clicking on academic words such as ‘cancer’ and ´oxygen´. Furthermore, the educational material is now easier to differentiate according to the students´ academic level [19].

Based on the process evaluation from the first evaluation of X:IT, the homepage (http://www.op-i-roeg.dk/) has been revised to meet some of the requests from the teachers, e.g. online teaching has been made available. Further, theme packages have been developed as they may require involvement from fewer teachers, an issue which was addressed in the first evaluation. The educational component of the intervention may still be taken on by one or more teachers from each class, according to the wishes and opportunities of each school. Schools can decide whether lessons are delivered during the whole school year or in special project days.

An overview of all intervention components in the X:IT II intervention is presented in Table 1. The table shows the main intervention components and any additional intervention activities. The table contains information on timing of all the activities including learning objectives for each activity.

**Table 1:** Intervention components of the X:IT II intervention, including timing of activities and learning objectives for each activity

	Setting	Intervention component	Timing	Aims
Main intervention components	School	Smoke free school time	Throughout the study period	- Remove exposure to smoking
				- Increase identification with non-smokers
				- Contribute to creating smoke-free environments
		Parent-teacher meetings	Start of every school term	- Present study information, especially the smoke free agreement
	Class	Smoke free curriculum	At least 8 lessons each year in year 7, 8 and 9	- Increase awareness of long- and short-term risks of smoking
				- Reduce majority misunderstanding
				- Increase awareness of smoking inducing mechanisms in society
				- Increase individual ability to resist temptation to smoke
				- Increase identification with non-smokers
				- Contribute to creating smoke-free environments
	Home/parents	Smoke free agreements and chat on tobacco	Start of every school term	- Create supportive smoke free environment at home
				- Signal opposition to adolescent smoking
				- Reduce availability of cigarettes
				- Reduce exposure to smoking
				- Increase identification with non-smokers
				- Contribute to creating smoke-free environments
Implementation and sustainability	School	Kick-off workshops for school coordinators and leaders	Before start of study, August 2017	- Inform about background and methods of the study
		Newsletters for schools	3–4 each year throughout study period	- Inform about study
				- Ensure sustainability of the study
		Study reports for each school	Fall 2018	- Inform about prevalences from study
				- Ensure sustainability of the study

### Hypotheses

Based on these modifications of the X:IT intervention, we hypothesize that:

- the X:IT intervention material, carefully developed to take into account the intellectual skills and academic performance of adolescents´ and their parents´, will improve the reach of the intervention among low socioeconomic background.
- the X:IT intervention study will have an overall effect of a 25% reduction in smoking uptake at age 13.
- the X:IT intervention study will reduce smoking uptake among low socioeconomic position adolescents to the same extent or more than among adolescents with a high socioeconomic position background.

### Theory

The X:IT II intervention is based on the theoretical framework of the X:IT study (the Theory of Triadic Influence) and is as such not applying specific new theories addressing social inequality in smoking. The results from the qualitative process evaluation of X:IT suggested that the practical application of the theoretical methods was inaccessible for children and parents from low SEP. The theoretical framework of the study is based on these findings and the fact that the X:IT interventions are population wide and meant to reach adolescents across socioeconomic positions.

### Evaluation

#### Design

The X:IT II intervention will be evaluated by means of effect in a difference-in-difference design. Hence, all participating schools will receive the same intervention, and students from high and low social classes will be compared in the difference-in-difference design [32]. Process evaluation will be conducted at specific time points during the intervention period. Data collections are shown in Table 2.

**Table 2:** Data collection of the X:IT II study – timeline, instrument and source of data

	Baseline September 2018	Intervention	Follow-up 1 May 2018	Intervention	Follow-up 2 April 2019	Intervention	Follow-up 3 April 2020
Students	Questionnaires (students start year 7)		Questionnaires (students end year 7)	Observations and interviews	Questionnaires (students end year 8)		Questionnaires (students end year 9)
School coordinators	Questionnaires		Questionnaires		Questionnaires		Questionnaires
Teachers		Monthly questionnaires on curricular activities (September – June)		Observations and interviews Monthly questionnaires on curricular activities (September – June)		Monthly questionnaires on curricular activities (September – June)	

#### Recruitment plan and study population

The recruitment period ran from January 2017 till June 2017, the intervention started in September 2017 and runs until June 2020. The recruitment ended up having two steps. First, 300 schools were randomly selected and contacted by email and by postal letter, followed up by telephone calls. The aim of this first step was to recruit 156 schools, which could be randomly selected into an intervention and a control group, i.e. to conduct a randomized controlled trial.

However, after contacting each of the 300 schools by telephone, it became clear that this aim was too ambitious, as only 60 schools showed any interest to be included in the study. In the second step of the recruitment phase, we decided to include all interested schools as intervention schools. However, three of these schools were for children with special needs and were therefore excluded from the evaluation, leaving 57 schools. After the summer holiday, when the final commitment from schools were needed and the data collection was about to start, 11 of the schools withdrew from the evaluation; in two schools, there was a new school leader who did not prioritize the intervention; two schools had assigned to too many projects and had to prioritize; at the remaining seven schools lack of time was the primary reason for resigning. Therefore, baseline data included 46 schools from all over Denmark.

#### Power calculations

Power calculations were based on the following assumptions: Each school cluster includes on average 50 students, the intra class coefficient for current smoking among grade 9 students is 0.053, smoking prevalence of 17.9% in grade 9, an expected reduction in smoking of 25 to 13.4%, and a power of 80%. Power calculations were conducted according to Donner & Klar (1996) [33] and showed a need for 48 schools with approximately 2400 students.

#### Implementation

Implementation was launched by a one-day-kick off workshop for school coordinators and leaders in the beginning of the school year 2017/2018. Representatives from the municipalities were also invited. The program for the day was planned and presented by staff from the Danish Cancer Society who developed and revised the intervention and the teaching materials, as well as staff from the Centre for Intervention Research in charge of evaluating the intervention.

The kick-off workshop included a detailed presentation of the intervention materials, the aim and background for the study, results from the evaluation of X:IT, and the rationale for the X:IT II intervention and evaluation. There was also room for reflection and chatting in smaller groups about experiences and expectations about implementing the intervention components. A folder with all background information about the curricular activities and good advises for implementation was given to the participants.

Parents were informed about the study and their involvement by two means: 1) at meetings for parents at each school in the beginning of the school year, 2) as written information on the actual smoke free agreement that students brought home. Students were informed about the study by the school coordinator at the school, and they received written information designed specifically for them.

### Effect evaluation

The effect evaluation will be based on quantitative data from questionnaires answered by students and project coordinators at schools. We will measure smoking status, sociodemographic factors including socioeconomic position of the family, and covariates such as parental and friend’s smoking at baseline.

### Primary outcomes

1. Frequency of smokers at grade seven, eight and nine measured by student self-reported questionnaires.
2. Degree of implementation after first, second and third year of intervention by student self-reported questionnaires and project coordinator questionnaires.

Measurements of the main variables are described in Table 3.

**Table 3:** Main variables measured in the X:IT study

Variables	Source of data	Items	Response categories	Dichotomizations and coding
Frequency of student smoking	Student self-reports	“How often do you smoke at present?”	Every day; not every day, but every week; not every week, but every month; more seldom than every month; never	Smokers are defined by ever smoking vs. never
Socioeconomic position	Student self-reports	“Does your father (mother) have at job?”	Yes; no; don’t know; don’t have/see my father (mother)	Information is coded into categories 1 (high) to 5 (low) according to the standards of the Danish National Research Institute of Social Research. Categories 6 (unclassifiable) and 7 (economically inactive) are added.
		“Why doesn’t he (she) have a job?”	He (she) is: sick; a student; seeking work; retired; staying at home; don’t know	
				Family social class is coded at the highest ranking parent
Degree of implementation (Adherence, dose, quality of delivery, participant responsiveness)^a^	School coordinator reports	(Adherence): “Are pupils allowed to smoke during school hours?”	Yes; yes outside school grounds; no	Adherence to the rules for smoke free school time are fulfilled if the school coordinator responds “no”
	Student self-reports	(Dose): “How often do you see students smoke different places at school?”	Daily; sometimes; never	Dose are fulfilled if students report never seeing other students smoking at school

^a^All measures of implementation are described in detail in Bast et al. 2016

#### Data collections

For the baseline study, data collection among students at the beginning of year 7 (age 13) and school coordinators are collected in the autumn 2017. Follow-ups are collected three times: at the end of year 7, 8 and 9 (age 15). Students are followed by using information on their name, birthday, class and school. This information is used for following students over time only. All further work with the data is in a non-identifiable version. Students changing to a school outside the evaluation are omitted from the study. Information about signed smoke free agreements is collected through the student questionnaires in each follow-up. To gain information about the curricular activities, school coordinators are asked to answer few monthly questions by email. Enforcement of the smoke free school time is measured by questions to students and to school coordinators in all questionnaires.

Students answer internet based questionnaires in the classroom after a standardized instruction given by the teacher. The students are informed that participation is voluntary and that their responses will be treated with confidentiality.

### Process evaluation

As was the case for the evaluation of X:IT [19–21, 25], we will conduct a detailed process evaluation among teachers and students focusing especially on those from low SEP. The process evaluation is based on questions added to the student and school coordinator questionnaires concerning the implementation of the study, e.g. the fidelity and dose of the intervention. Further, we will conduct ethnographic fieldwork at schools and qualitative interviews with students, school coordinators and other teachers involved in X:IT II. Information about the actual curricular activities is collected through monthly electronic questionnaires to teachers.

As the quality of implementation has a great impact on the conceivable effects, the process evaluation will have a specific focus on implementation. The organizational capacity for implementation at each school will be assessed through the baseline questionnaires for school coordinators. These measures are based on theories by Domitrovich et al. (2008) [34] and Saccia et al. (2015) [35], hence we will measure Motivation (i.e. the perceived relative advantage of X:IT compared to other programs, the complexity of X:IT and the priority for the school), General organizational capabilities (i.e. school culture, climate and leadership), and Innovation-specific capabilities (i.e. knowledge, skills and abilities needed for the intervention and the presence of a program champion). Further, the actual obtained implementation will be assessed at all follow-ups by both school coordinators and students, based on implementation measures developed in the evaluation of X:IT [20].

The qualitative data will focus on the school coordinator and teacher’s implementation of the X:IT II intervention. Thus, we will explore: 1) the teacher’s appraisal and reaction to the intervention and activities, 2) how the teachers experience and understand the problem that the intervention seeks to address, and 3) how the intervention activities are implemented in practice [36–38]. To understand adolescent’s smoking related behavior, we will investigate the influence of adolescent’s social relations and smoking in the daily environment [39, 40]. This will help us understand how the students perceive and receive the intervention.

The ethnographic field study (comprising observations and interviews) will provide in-depth knowledge about contextual circumstances regarding the implementation, delivery and reception of the intervention. These methods, will enable us to account for the context in which events occur and uncover social patterns, for example, which relationships are important for actions related to the X:IT intervention and hence smoking [41]. By being physically present at schools, taking part in and observing the students and teachers carrying out their daily activities, we will be able to understand how the intervention is practiced at schools and how it and smoking is perceived by students and teachers [41].

### Statistical methods

#### Data process and analyses

The analyses of effect will be based on the principles of the difference-in-difference approach. The difference-in-difference design compares outcomes after and before an intervention between an exposure group and a comparison group [32]. In the evaluation of X:IT II, we will consider the group of students with high SEP as the comparison group (group C) and students with low SEP as the exposed group (group E). This means that the change in outcomes can be estimated as (Cafter-Cbefore) - (Eafter-Ebefore). If X:IT II works equally well for students from both high and low SEP, the difference-in-difference estimate is equal to 0 which is the aim of X:IT II.

The difference-in-difference estimates will be conducted in regression models rather than by simple subtraction which allows adjusting for confounders. We will conduct both per-protocol analyses and intention-to-treat (ITT) analyses using multiple imputation of missing values. Furthermore, we will use statistical methods that take into account the multilevel structure of the design of the study.

## Discussion

The primary focus of this study is to evaluate the smoking preventive intervention – X:IT II. We expect the revised version of the X:IT intervention to be equally successful in preventing smoking uptake in adolescents from high and low socioeconomic backgrounds. Smoking prevalence is higher among low SEP adolescents [1] and contributes to a large extent to the social inequality in health over the lifetime [5].

Public health interventions are often accused of leading to higher social inequality when implemented across populations [4]. School-based interventions to prevent smoking can also widen this inequality, however studies have also been found to reduce the gap; secondary analyses of three overall effective school-based European smoking interventions showed mixed results. One seemed to widen socioeconomic inequality, while the two other studies showed mixed results depending on which measure of socioeconomic status that were used [42]. One of the conclusions from the study was that adolescent smoking is also influenced by factors outside the school, especially smoking by parents and other significant relations, which are important to include when aiming at reducing the social inequality in intervention effects [42]. The changes conducted in the X:IT II intervention are based on thorough interview work with the target population of children from low social class and their parents. The changes should therefore be highly relevant and target some of the specific problems related to a smoking preventive intervention, such as the X:IT intervention.

The X:IT II intervention is based on an intervention which has been thoroughly evaluated and has proven effective, especially when implemented correctly. The X:IT II intervention target adolescent smoking from multiple levels simultaneously; the individual level, the family level, and the school level. This is recommended by the literature and must be considered state of the art in school-based smoking interventions [11]. The X:IT intervention is developed and adjusted by the Danish Cancer Society and will be evaluated by the Centre for Intervention Research. Especially in an intervention which is not a randomized controlled trial it is of great value, that the evaluating organization is independent from the organization that developed and implemented the intervention.

One of the limitations of this study is that we did not succeed in recruiting the required number of schools for a randomized design. The clustering of smoking in schools and classes leads to the need of a very high number of schools for an RCT, in this case 78 schools for intervention and 78 for comparison. The main purpose of the X:IT II study is to examine the differences between high and low SEP and the mechanisms behind these differences. The difference-in-difference design suits this purpose. There are two main assumptions to the difference-in-difference design, the assumption of parallel trends and the assumption of common shocks. First, the parallel trends assumption states that trends in outcomes between the exposed and comparison groups are parallel prior to the intervention. As we know from the literature, that smoking is more prevalent among both adolescents and adults from lower SEP [1, 43], it is not possible to fulfill this assumption. On the other hand, at the beginning of grade 7, very few students have become smokers and as such a trend in smoking is not calculable in this age group. We expect that the second assumption of common shocks stating that any events occurring during or after the intervention will equally affect the exposure and comparison groups, will be fulfilled. Power calculations for the difference-in-difference design showed a need for 48 schools, which was fulfilled in the recruitment where 57 schools gave consent to participate. However, as previously described only 46 schools participated in the baseline data collection.

To further improve the evidence from this study, a thorough process evaluation is planned including both qualitative and quantitative methods. The qualitative evaluation will seek to understand how teachers work with the intervention and under which circumstances the best implementation is obtained. Further, we will obtain knowledge and understanding on how students perceive and receive the intervention. The quantitative process evaluation will, as was the case in the former evaluation of X:IT, be based on students receiving the intervention. Also, school capacity to implement, as well as the actual obtained implementation, will be assessed. Like in the X:IT study [20, 25], we will conduct analyses taking the actual implementation into account in the effect evaluation. Hereby, we will be able to draw conclusions about the effectiveness of the X:IT evaluation. We will examine intervention effects in relation to the actual implementation at the school as well as the individual level. Numbers of students receiving the intervention in X:IT II is almost equal to the group of intervention pupils from the first evaluation. Analyses will therefore be comparable to each other.

If shown to be effective, the X:IT II study will provide important information on how to prevent smoking uptake effectively also among the group of adolescents from low socioeconomic backgrounds who are currently at higher risk of smoking uptake in Denmark.

## Abbreviations

RCT: randomized controlled trial
SEP: socioeconomic position
